# Involvement of C4 Protein of Beet Severe Curly Top Virus (Family *Geminiviridae*) in Virus Movement

**DOI:** 10.1371/journal.pone.0011280

**Published:** 2010-06-24

**Authors:** Kunling Teng, Hao Chen, Jianbin Lai, Zhonghui Zhang, Yuanyuan Fang, Ran Xia, Xueping Zhou, Huishan Guo, Qi Xie

**Affiliations:** 1 State Key Laboratory of Plant Genomics and National Center for Plant Gene Research, Institute of Genetics and Developmental Biology, Chinese Academy of Sciences, Beijing, China; 2 State Key Laboratory of Plant Genomics and National Center for Plant Gene Research, Institute of Microbiology, Chinese Academy of Sciences, Beijing, China; 3 State Key Laboratory of Rice Biology, Institute of Biotechnology, Zhejiang University, Hangzhou, China; University of Heidelberg, Germany

## Abstract

**Background:**

Beet severe curly top virus (BSCTV) is a leafhopper transmitted geminivirus with a monopartite genome. C4 proteins encoded by geminivirus play an important role in virus/plant interaction.

**Methods and Findings:**

To understand the function of C4 encoded by BSCTV, two BSCTV mutants were constructed by introducing termination codons in ORF C4 without affecting the amino acids encoded by overlapping ORF Rep. BSCTV mutants containing disrupted ORF C4 retained the ability to replicate in Arabidopsis protoplasts and in the agro-inoculated leaf discs of *N. benthamiana*, suggesting C4 is not required for virus DNA replication. However, both mutants did not accumulate viral DNA in newly emerged leaves of inoculated *N. benthamiana* and Arabidopsis, and the inoculated plants were asymptomatic. We also showed that C4 expression in plant could help C4 deficient BSCTV mutants to move systemically. C4 was localized in the cytosol and the nucleus in both Arabidopsis protoplasts and *N. benthamiana* leaves and the protein appeared to bind viral DNA and ds/ssDNA nonspecifically, displaying novel DNA binding properties.

**Conclusions:**

Our results suggest that C4 protein in BSCTV is involved in symptom production and may facilitate virus movement instead of virus replication.

## Introduction

*Curtovirus* is one genus of the family *Geminiviridae*, a group of plant viruses with small DNA genomes containing one or two circular DNA components. Viruses from this genus are transmitted by leafhoppers and can infect a wide range of dicotyledonous plants, including many important crops such as sugar beet, common bean, tomato and pepper, primarily in the western United States [1]. The infected plants exhibit an economically important disease with symptoms including stunted growth, leaf curling, accumulation of anthocyanin, vein swelling and hyperplasia of the phloem [2]. To date, certain host factors in plants were identified to be involved in *Curtovirus* infection [3], [4], [5]. The genome of geminivirus is either monopartite or bipartite, while viruses in *Curtovirus* have a monopartite genome [6], [7], [8], [9], and their infection in plants is phloem-limited [10], [11]. They have been grouped into five species: *Beet curly top virus* (BCTV, formerly California/Logan strain), *Beet severe curly top virus* (BSCTV, formerly CFH strain), *Beet mild curly top virus* (BMCTV, formerly Worland strain), *Horseradish curly top virus* (HrCTV) and *Spinach curly top virus* (SpCTV) [12]. Recently, a new species, *Beet curly top Iran virus* (BCTIV), has been reported [13].

To infect the host successfully, geminiviruses must first replicate in the plant cell nucleus, then move from cell to cell, and finally move throughout the plant via phloem-mediated transport. In this process, geminiviruses must overcome two distinct barriers posed by the nuclear envelope and the plant cell wall to infect the plant systemically [14], [15]. Certain movement proteins encoded by the virus are involved in this process. Two different proteins have been identified to participate in virus movement in bipartite geminivirus, the movement protein (MP, encoded by the gene *BV1*) and the nuclear shuttle protein (NSP, encoded by the gene *BC1*)[16], [17]. NSP protein can bind ssDNA and dsDNA and has the ability to shuttle between the nucleus and the cytoplasm, while MP shuttles between the nuclear envelope and the cellular periphery [18], [19], [20], [21], [22]. MP and NSP protein in BDMV are both able to recognize DNA in a form- and size-specific manner and transport DNA from cell to cell [20], [23]. Coat protein (CP), which is essential for systemic infection in monopartite geminivirus [24], [25], does not appear to be absolutely necessary for this process [17], [26]. The “relay race model” and the “couple-skating model” have been proposed to describe the movement of the bipartite geminiviruses [20], [22], [23], [27], [28]. There are only a few reports demonstrating the possible mechanism of virus movement in monopartite geminiviruses. Except CP as an essential factor, V1 and C4 protein have also been implicated for monopartite geminivirus movement [25], [29], [30]. Rojas et al proposed a possible model for CP-mediated nuclear export of viral DNA and V1-mediated delivery of viral DNA to the cell periphery in a whitefly-transmitted monopartite begomovirus, *Tomato yellow leaf curl virus* (TYLCV). In their study, C4 localized at the cell periphery and had a limited capacity to mediate cell-to-cell movement of viral DNA [29].

C4 has been shown to be an important protein in virus/plant interactions while displaying diverse functions. In bipartite begomoviruses *African cassava mosaic virus* (ACMV) and *Sri Lankan cassava mosaic virus* (SLCMV), AC4, a homologue of BSCTV C4, can suppress post transcriptional gene silencing by binding miRNAs and siRNAs [31], [32]. Tomato leaf curl virus (ToLCV) C4 protein also acts as a gene silencing suppressor and interacts with a novel shaggy-like kinase (SISK) in a yeast two-hybrid screen [33]. However, AC4 in TGMV which is also bipartite begomoviruse showed either no function or redundant function with other TGMV encoded proteins [34]. Previous studies of BCTV showed that the open reading frame (ORF) C4 is responsible for symptom determination and mutations in C4 caused quite different symptoms compared to that caused by wild type C4 in host plants [35]. Meanwhile, expression of C4 protein in *N. benthamiana* and Arabidopsis produced abnormal cell division and altered plant development possibly by disruption of hormone pathways [2], [36]. Besides, C4 interaction with Arabidopsis AtSKη suggests that C4 may be involved in the brassinosteriod signaling pathway [3]. However, C4 protein involvement in virus movement has not been reported in *Curtovirus*.

Here, we present a functional analysis of C4 protein encoded by leafhopper transmitted *Curtovirus* BSCTV. Analysis of viral infectivity and DNA replication with BSCTV and C4 deficient BSCTV mutants revealed that BSCTV C4 is essential for disease symptom formation, but not required for viral DNA replication. Expression of BSCTV C4 in plants can rescue systemic movement of C4 deficient BSCTV mutants. Together with the non-specific ss/dsDNA binding activity of C4 and its cell nucleus and cytosol localization feature, our results suggest that BSCTV C4 is a nuclear shuttle protein that mediates movement of BSCTV DNA.in Arabidopsis and *N. benthamiana*.

## Results

### BSCTV C4 affects symptom formation in host plants

**Figure 1:**
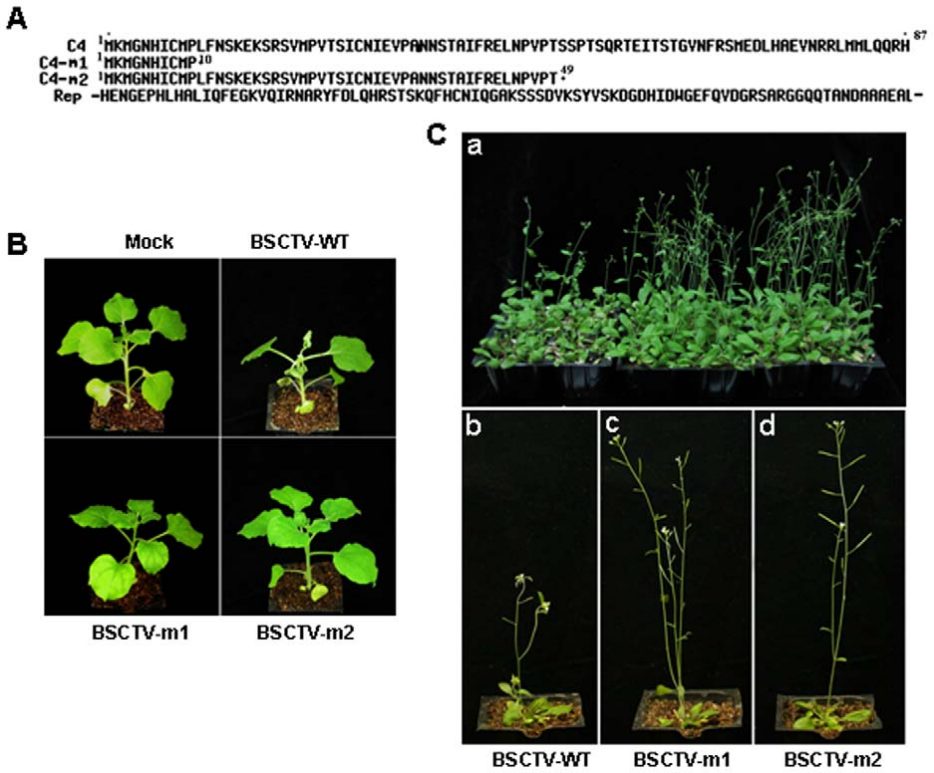
The position of two mutations in C4 protein and disease symptoms in *N. benthamiana* and Arabidopsis infected by BSCTV C4 mutants. (A) Amino acid sequences of C4 and two C4 mutants created by nucleotide substitution (see materials and methods). Numbers indicate the position of the first and the last amino acid. (B) *N. benthamiana* plants infected with wild-type and C4 mutated BSCTV two weeks after agro-inoculation. (C) Arabidopsis plants infected with wild-type and C4 mutated BSCTV two weeks after agro-inoculation. a shows 20 plants infected with wild-type, BSCTV-m1 and BSCTV-m2 respectively. b, c, d shows the individual plant infected with wild-type, BSCTV-m1 and BSCTV-m2 respectively.

BSCTV *C4* encodes a small protein composed of 87 amino acids, and little is known about the function of this protein in virus/plant interactions. To investigate the function of C4, we introduced two mutations in the *C4* ORF independently, producing two premature proteins containing the truncated 10 and 49 amino acids respectively. However, the nucleotide mutations of C4 had no effect on the amino acid sequence of the overlapping Rep protein which is required for the replication of viral DNA (Fig. 1A). Both Arabidopsis and *N. benthamiana* plants can be infected with wild-type BSCTV by agro-inoculation method (see Materials and Methods) and produced severe symptoms such as plant stunting, vein swelling and severe upward rolling of young leaves two weeks post-inoculation. The infection ratio was 100% (8/8 plants were infected; experiment repeated three times) in *N. benthamiana* and up to 95% in Arabidopsis, respectively (Fig. 1B and Fig. 1C). In contrast, all plants including *N. benthamiana* and Arabidopsis inoculated with both C4 deficient BSCTV mutants remained asymptomatic (Fig. 1B and Fig. 1C), even up to two months. This indicates that BSCTV C4 protein is essential for symptom formation in plants.

### Mutation of C4 protein impaired the viral DNA accumulation in newly emerged leaves in plants

**Figure 2:**
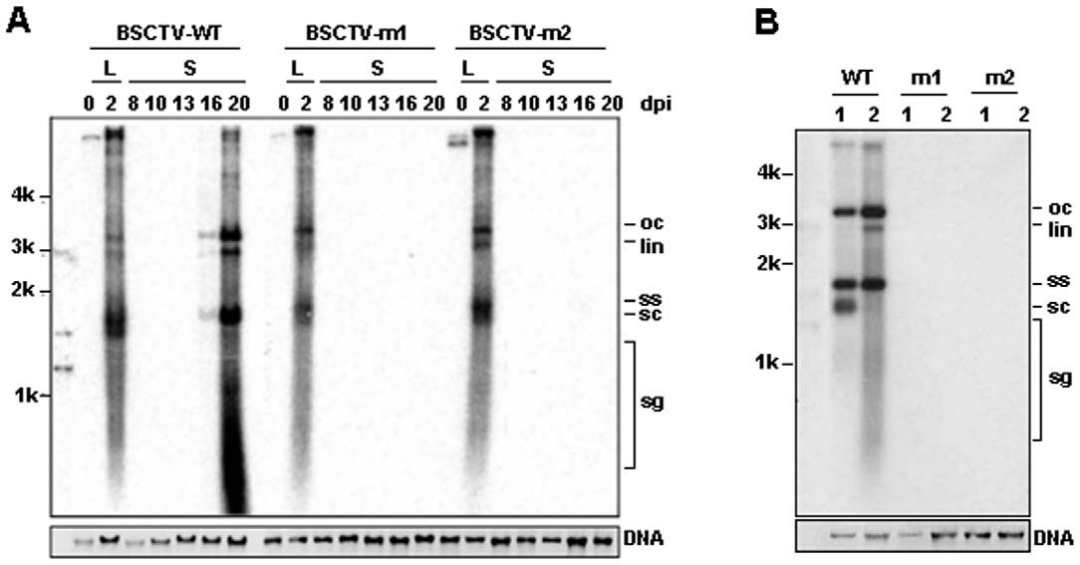
Viral DNA analysis in *N. benthamiana* and Arabidopsis plants agro-inoculated with wild-type and mutated BSCTV. (A) Southern blot analysis of total nucleic acids extracted from agro-inoculated (0 and 2d) and newly emerged (0, 2, 8, 10, 13, 16 and 20d) leaves of *N. benthamiana* after agro-inoculation. (B) Southern blot analysis of total nucleic acids extracted from newly emerged leaves of Arabidopsis two weeks after agro-inoculation. Size marker (the first lane on the left) was a mixture of BSCTV fragments digested by *Tth*111I and *Eco*RI, and the fragment digested by *Eco*RI. Fragment sizes are given in kb. The positions of open circle (oc), linear (lin), supercoiled (sc) and single stranded (ss) DNAs, and a population of subgenomic DNA forms are indicated.

After the agro-inoculation, viral genomes can be released from the tandem repeats and the replicative forms of viral DNA, such as double-stranded and single-stranded DNA, can then be detected in plant leaves. To discover why plants inoculated by C4 deficient BSCTV mutants remain asymptomatic, total DNA was extracted from the inoculated and newly emerged leaves of *N. benthamiana* to detect the presence of BSCTV viral DNA. DNA gel blot analysis for the DNA from the inoculated leaves of *N. benthamiana* at two days post inoculation revealed that accumulation of mutant viral DNA was similar to that of wild-type (Fig. 2A). This result indicates that both C4 mutations in BSCTV genome could not impair the virus capability to infect the inoculated leaves of *N. benthamiana* plants. However, viral DNA was not detected in newly emerged leaves of *N. benthamiana* inoculated with C4 deficient BSCTV mutants, while inoculated with wild-type virus, it was easily detected 16 and 20 days post inoculation (Fig. 2A). Viral DNA of C4 deficient mutants were not detected in newly emerged leaves even two months later (data not shown). Similar results were obtained in the newly emerged leaves of Arabidopsis two weeks post inoculation (Fig. 2B), and the mutated viral DNA was also not detected by high sensitive PCR detection method (Data not shown). These results indicate that C4 mutation impaired the viral DNA accumulation in newly emerged leaves in plants.

### C4 mutation does not impair virus replication

**Figure 3:**
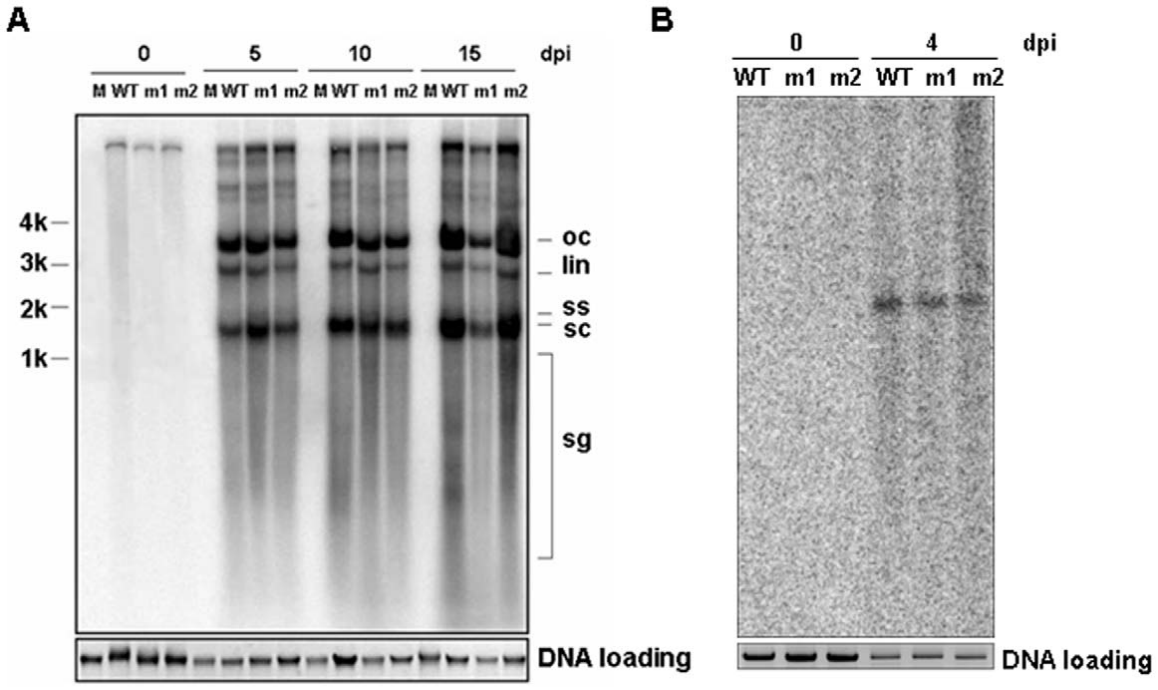
Viral DNA analysis in *N. benthamiana* leaf discs and Arabidopsis protoplasts infected with wild-type and mutated BSCTV. (A) Southern blot analysis of total nucleic acids extracted from leaf discs at 0, 5, 10, 15d after agro-inoculated with wild-type and mutated BSCTV. (B) Southern blot analysis of total nucleic acids extracted from Arabidopsis protoplasts at 0 and 4d after transfection of plasmids containing wild-type and mutated BSCTV.

The impairment of viral DNA accumulation in plant newly emerged leaves may be caused by either the impairment of viral DNA replication or the restricted spread of virus movement from agro-inoculated leaves to newly emerged leaves. Therefore, the capability of the C4 deficient BSCTV mutants to replicate was investigated by *N. benthamiana* leaf disc assay. Total DNAs were extracted from the leaf discs agro-inoculated with wild-type and C4 deficient BSCTV at 5, 10 and 15 days post inoculation. DNA gel blot analysis showed that both the mutant viral DNA accumulated to a similar level to wild-type viral DNA (Fig. 3A). The ability of wild-type and C4 deficient BSCTV to replicate in single plant cells was also investigated by transfecting Arabidopsis protoplasts with the respective infectious clones. Total DNA were extracted from the protoplasts 4 days after the transfection and DNA gel blot analysis showed that mutant viral DNA accumulation was also similar to that of wild-type (Fig. 3B). Together, these results demonstrate that the mutations in C4 protein do not affect viral DNA replication.

### Expression of C4 protein in plants facilitates the BSCTV mutant to move systemically

**Figure 4:**
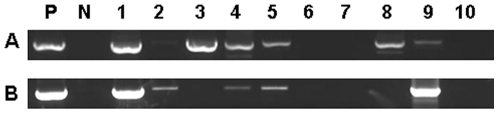
Diagnostic PCR analysis of BSCTV in infected newly emerged leaves of C4 transgenic Arabidopsis. Genomic DNAs from each of 10 individual plants infected with BSCTV-m1 (A) and BSCTV-m2 (B) were used as templates. P indicates positive control (infected with wild-type BSCTV) and N indicates negative control (not infected with virus). Lane 1 shows DNA from the plants produced symptoms, and lanes 2–10 show DNA from plants without symptoms.

As C4 mutation does not affect viral DNA replication, it is possible that this mutation restricts the movement of virus from the inoculated leaves to the newly emerged leaves. To explore this possibility, C4 transgenic plants in which C4 expression was controlled under an inducible promoter in pER8 vector [4], [37] was inoculated with both BSCTV-m1 and BSCTV-m2 mutants (40 for each) and *C4* expression were induced immediately by watering solution containing the inducer β-estradiol. Total DNA was extracted from the newly emerged leaves of 20 randomly selected plants (10 for each mutant) three weeks post inoculation. A 1065 bp fragment of BSCTV genomic DNA covering the full length Rep ORF that overlapping ORF C4 was detected by PCR analysis. Detection of this fragment could reflect the presence of viral DNA and avoid the false positive detection of the transgenic *C4* gene. As shown in Fig. 4, the viral DNA fragment could be detected in 5 plants inoculated with BSCTV-m1 and 4 plants inoculated with BSCTV-m2. We sequenced all the DNA fragments obtained from PCR and found that all the fragments contained the relevant mutated C4 sequence except two containing wild-type C4 sequence. These results demonstrate that expression of C4 protein could rescue the movement of C4 deficient mutants in plants. The presence of wild-type C4 may be caused by recombination between the C4 transgene and the mutated C4 gene within the virus genome as CP acts in ACMV [38].

### C4 can bind dsDNA and ssDNA nonspecifically

**Figure 5:**
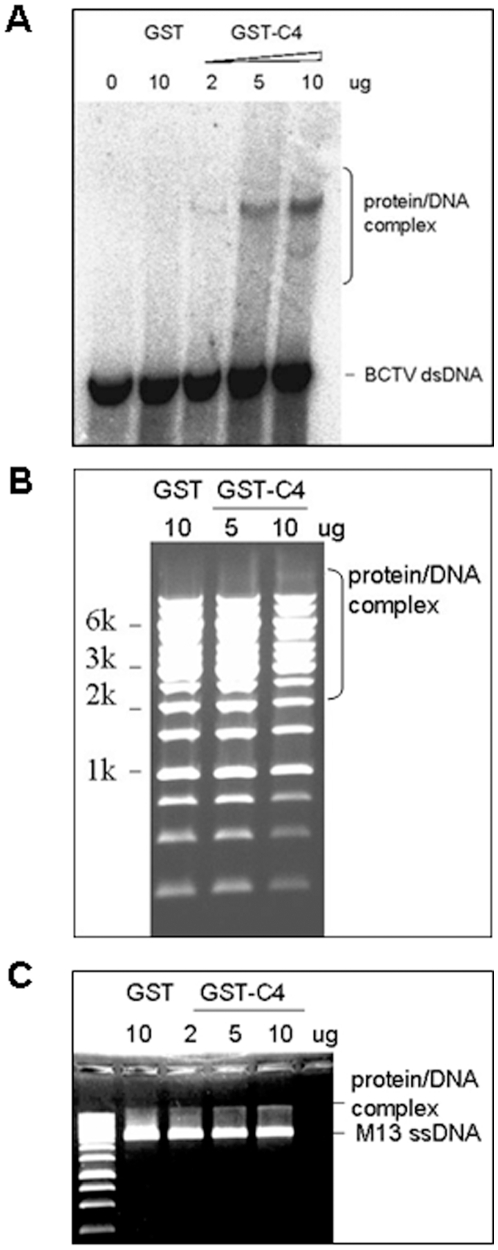
Binding affinity of C4 protein with dsDNA and ssDNA. (A) Protein-DNA binding assay for C4 binding double strand BSCTV DNA. BSCTV fragments digested by *Eco*RI were end-labeled with [α- ^32^P]dATP and Klenow polynucleotide kinase as a probe. Increasing amounts of C4 proteins (2–5 µg) were incubated with 50 ng probes at 22°C for 30 min. Gels were dried and the migration of labeled DNA was detected by a PhosphorImager. (B) Increasing amounts of C4 protein mixed with 0.5 µg 1 kb linear dsDNA ladder. DNAs were detected by visualization of ethidium bromide stained gels. C) Increasing amounts of C4 protein mixed with 0.2 µg M13 ssDNA. DNAs were detected by visualization of ethidium bromide stained gels. All mixtures were analyzed in 0.7% nondenaturing agarose gels in TBE buffer. The protein/DNA complexes are indicated.

Viral proteins involved in geminivirus movement may bind virus particles, virus DNA and/or other viral proteins. Previous studies demonstrated that some viral proteins involved in DNA virus movement had the capacity to bind DNA [20], [22]. Our results indicate that C4 appears to be involved in virus movement; but its ability to bind ss and/or dsDNA was to be evaluated so far. By bioinformatic analysis, the probability of C4 protein binding to DNA predicted by sequence analysis (http://www.netasa.org/dbs-pred/) was 95.8%. Thus, the protein-DNA binding assay was performed using linear monomer of BSCTV genomic DNA excised from the plasmid pCambia-1300-BSCTV. The C4 protein was fused to the GST tag, expressed in *E. coli* and purified for this assay, while GST protein itself was also expressed and purified as an internal control. It was shown that the mobility of BSCTV dsDNA was retarded in the presence of C4 protein (Fig. 5A), and the presence of the protein/DNA complex was increased with elevated C4 protein concentration during incubation. This result establishes that C4 could bind BSCTV viral DNA. In addition, the DNA binding ability of C4 protein was proved to be unspecific by protein-DNA binding assay using a 1 kb DNA ladder of linear dsDNA fragments. Figure 5B shows the protein/DNA complex and highlights the relationship between increasing concentrations of C4 and the amount of the retarded DNA fragments. The ability of C4 to bind ssDNA was also verified by a similar assay using a commercial source of M13 mp 18 circular ssDNA (GE healthcare) (Fig. 5C). The above results indicated BSCTV C4 can bind both its own viral DNA and non specific ss/dsDNA.

### Localization of C4 protein in Arabidopsis protoplasts and *N. benthamiana* leaves

**Figure 6:**
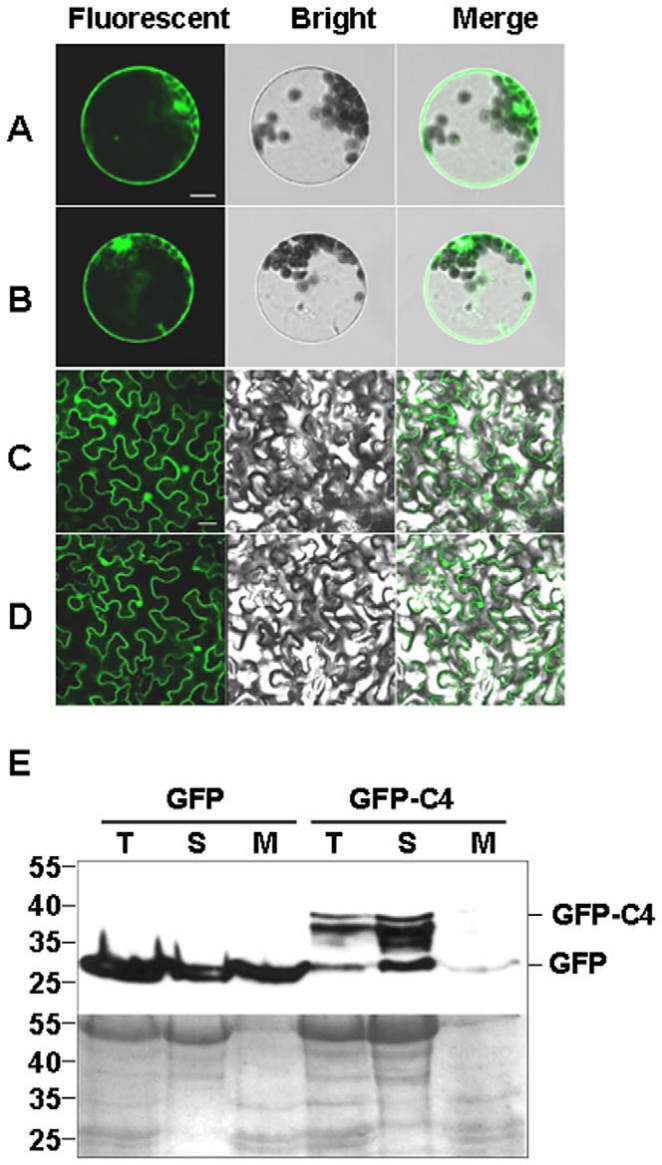
Subcellular localization of C4 protein. A) The fluorescence observation of control GFP protein in Arabidopsis protoplasts. Bar  = 10 µm. (B) The fluorescence observation of GFP-C4 fusion protein in Arabidopsis protoplasts. Bar is common to (A). (C) The fluorescence observation of control GFP protein in *N. benthamiana* leaves. Bar  = 20 µm. (D) The fluorescence observation of GFP-C4 fusion protein in *N. benthamiana* leaves. Bar is common to (C). Cells were analyzed by confocol microscopy. F indicates fluorescence, B indicates bright light and M indicates merged. (E) Cell fraction assays of GFP and GFP-C4 fusion protein. Total extract of *N. benthamiana* leaf cells expressing a GFP control and GFP-C4 fusion proteins were fractionated into soluble (S) and microsomal (M) fractions. GFP and GFP-C4 fusion proteins were detected using a anti-GFP antibody and indicated (top panel). Ponceau S staining of the transferred membrane is displayed as a loading control (bottom panel).

Since C4 could bind viral DNA, the localization of C4 protein in plant cells may provide us a clue to investigate the role of C4 protein in BSCTV movement. To find out its localization, C4 ORF were fused to the 3' end of the *GFP* gene and expressed under the control of the 35S promoter, generating two constructs pGFP2-C4 and pBAL-GFP-C4, transient expression and transgenic expression, respectively. The transient expression construct pGFP2-C4 was transfected into Arabidopsis protoplasts for transient expression. GFP fluorescence was observed 16 h after the transfection using a confocol laser scanning microscope. GFP fused C4 protein was found both in the cytoplasm and the nucleus, similar to the expression of the GFP control (Fig. 6A, 6B) Transient expression of GFP fused C4 protein in *N. benthamiana* leaves by agro-infiltrated pBAL-C4-GFP showed the similar localization (Fig. 6C, 6D). However, these results of C4-GFP fusion could not distinguish whether C4 protein was also localized to the cell membrane, thus cell fraction assay was performed. Total proteins, soluble proteins and microsomal fractions were extracted from *N. benthamiana* leaves agro-infiltrated by pBAL-GFP and pBAL-C4-GFP, respectively. Protein gel blot analysis using GFP antibodies showed that GFP protein was detected both in soluble and microsomal fractions while the GFP-C4 fusion protein was detected exclusively in the soluble fraction and not in the membrane fraction (Fig. 6E). Taken together, these results demonstrate that C4 is localized in cell nucleus and cytosol, but not in cell membrane, indicate C4 may facility virus movement between cell nucleus and cytosol.

## Discussion

In this study, we have shown that plants infected with C4 deficient BSCTV mutants, which contain premature C4 but not alter the amino acid sequence of the overlapping Rep protein, developed asymptomatic phenotype (Fig. 1). The fact that viral DNA replication of C4 deficient BSCTV mutants equaled well to that of wild type BSCTV in agro-inoculated leaves and protoplasts (Fig. 3), but not in newly emerged leaves of infected plants (Fig.2), indicating that the C4 deficient BSCTV mutants lack the capacity of viral DNA movement rather than replication. Our results indicate that BSCTV C4 may play an important role in virus movement rather than replication in host plants. Induced expression of BSCTV C4 in transgenic Arabidopsis can rescue systemic movement of C4 deficient BSCTV mutants (Fig. 4), further supporting that BSCTV C4 functions as a movement protein to mediate BSCTV viral DNA transport.

To achieve long distance movement, geminiviruses which DNA replication happens in the nucleus must first move out from nucleus to cytosol, then move from cell to cell, and finally throughout the plant via phloem-mediated transport. In our DNA binding assay, BSCTV C4 was shown to bind both dsDNA and ssDNA *in vitro*, which provide us a clue that BSCTV may bind its own viral DNA and may mediate viral DNA transport in certain stages of virus movement. Besides, due to its nonspecific DNA binding activity, we could not exclude the possibility that C4 may bind certain non-viral DNA, such as host genomic DNA, and play other roles in the virus infection. Our cell fraction assay in *N. benthamiana* leaves and the subcellular localization of GFP-C4 fusion protein indicated that C4 protein of BSCTV localizes in cell nucleus and cytosol, rather than associates with cell membrane. This result suggests that BSCTV C4 is a nuclear shuttle protein and may mediate viral DNA to move between the nuclear and cytoplasm. We can not rule out the possibility that BSCTV C4 may also involve in viral DNA cell to cell movement. Previous studies on *Curtovirus* described that C4 encoded by TYLCV was found to localize to the cell periphery, and suggested to mediate viral DNA to move from cell to cell in phloem cells [29]. BCTV C4 protein expression in *N. benthamiana* leaves was detected at cell periphery and a low level in the nucleus, and suggested to associate with plasma membrane [3]. Meanwhile, CP encoded by BMCTV was reported to mediate long distance movement of virus [39]. Taken together, these results suggest that C4 encoded by monopartite *Curtovirus* function as movement protein as MP and NSP proteins in bipartite geminivirus [23], [27], [28], [40] to mediate viral DNA to shuttle between the nucleus and the cytoplasm and/or between the nuclear envelope and the cellular periphery.

There was no obvious disease symptom in BSCTV mutants infected C4-induced-expressing plants, in which BSCTV mutants DNA detected in newly emerged leaves (Fig. 4), probably due to the low amount of expressing C4 protein in transgenic plants induced by watering inducer containing solution. This was supported by appearance of symptom-like phenotype when C4 transgenic plants were continuously applied inducer to keep expressing BSCTV C4 (Teng and Xie, unpublished data). Together with our previous study that constitutive expression of BSCTV C4 induced abnormal cell division/differentiation, and that normal transgenic lines could not be generated (4), our results suggest that symptom determination is other biological functions of BSCTV C4. In other two monopartite geminiviruses studies, mutations in BCTV C4 result in different symptoms development from that caused by wild-type BCTV [8]. Similarly, a TYLCV-C4stop mutant produced weak symptoms and reduced viral DNA in *N. benthamiana* compared to wild-type virus [30]. However, mutations in the C4 homologue in bipartite geminiviruses have no obvious effect on symptom development or infectivity [34], [41], [42]. Taken together, it is likely that C4 is essential for symptoms production in monopartite *Curtovirus* but not in bipartite geminiviruses.

In summary, we demonstrated that the C4 protein of *Curtovirus* BSCTV, plays an important role in virus movement in Arabidopsis and *N. benthamiana*. Localization of BSCTV C4 protein and its DNA binding activity suggest that BSCTV C4 function as a nuclear shuttle protein to bind viral DNA and mediate the movement of virus between nucleus and cytosol. BSCTV C4 is also essential for disease symptoms formation. It is not surprising in view of many geminivirus encoded proteins appearing to be multifunctional [15]. The precise mechanism of C4 in virus movement and other cellular processes needs to be revealed in the future.

## Materials and Methods

### Construction of viral clones for agro-inoculation

Agro-inoculation was used to analyze the infectivity of the cloned virus DNA. An infectious clone pCFH containing the full length genomic DNA of BSCTV (ATCC number: PVMC-6) was obtained from American Type Culture Collection (ATCC, Manassas, VA). First, a 536 bp *Bam*HI-*Eco*RI fragment from pCFH as a 0.2 copy of BSCTV genome was inserted into a binary plant transformation vector pCambia-1300 (CAMBIA, Canberra, Australia), generating plasmid pCambia-1300/BSCTV-0.2. Subsequently, a complete genome unit of BSCTV, excised with *Eco*RI from pCFH infectious clone, was then cloned into the linearized pCambia-1300/BSCTV-0.2 to generate pCambia-1300-BSCTV containing the partial tandem repeat harboring 1.2 copy of BSCTV genome.

Site-directed mutagenesis was done using pUC8 containing the whole BSCTV genome inserted at the *Eco*RI unique site (reverse) with the Quick Change Site-Directed Mutagenesis Kit (Strategene, CA, USA). Two independent changes were introduced into pUC8-BSCTV. The first, a T to A mutation at position 2386 in BSCTV genome, created a premature stop codon TAA, truncating the C4 protein after 10 amino acids. The second, a C to A mutation at position 2503 in BSCTV genome, also created a premature stop codon TAA that truncated the C4 protein after 49 amino acids. The two mutated whole genome BSCTV units were cloned into pCambia-1300/BSCTV-0.2, generating plasmids pCambia-1300-BSCTV-m1 and pCambia-1300-BSCTV-m2, respectively. All constructs were introduced into *Agrobacterium tumefaciens* strain EHA105 [43]. *Nicotiana benthamiana* and *Arabidopsis thaliana* plants were agro-inoculated as described below.

### Agro-inoculation of viral clones to Arabidopsis and *N. benthamiana*

Agrobacterium containing the viral clones were grown at 28°C overnight and resuspended in 10 mM MgCl_2_ solution adding 150 µM acetosyringone to a final concentration of OD_600_ = 2.0. The suspensions were kept at room temperature for 3–5 hours without shaking. After mixed with 1% carborundum (320 grit, C192-500, Fisher Scientific), 4-week-old Arabidopsis plants were agro-inoculated using airbrush technique as [44] described with an air pressure of 75 to 80 psi during spraying. One leaf of each 3-week-old *N. benthamiana* plants was wounded by rubbing with carborundum through the *Agrobacterium* inoculum.

### DNA gel blot analysis

For DNA gel blot analysis, genomic DNA was isolated using a CTAB buffer. Total genomic DNA (1 µg) was separated by electrophoresis in 0.8% agarose gels and transferred to Hybond N+ membrane (Amersham Pharmacia Biotech). The specific probe for DNA gel blot was BSCTV whole genome digested from pCambia-1300-BSCTV with *Eco*RI. The specific probes were labeled with [α-^32^P]dCTP using a Ready-primed labeling kit (Amersham International). DNA accumulation was detected using a PhosphorImager (Bio-Rad, Hercules, CA).

### Protein expression and purification

C4 coding sequence was cloned to plasmid pGEX-6P-1 and transformed into *Escherichia coli* XL1-blue strain. When the cells reached a density of OD_600_ = 0.6–0.8, the GST-C4 fusion protein was induced with 0.4 mM IPTG at 18°C overnight. The cells were collected and resuspended in GST binding buffer (50 mM Tris-HCl at pH 8.0; 200 mM NaCl; 1 mM EDTA; 1% NP40; 1 mM DTT and 1 mM PMSF), and lysed by sonication (5×20 s) on ice. The GST-C4 fusion protein was purified by Glutathione Sepharose 4B beads (GE healthcare), and washed with 10 mM reduced glutathione. GST protein as a control was expressed and purified in the same way.

### Protein-DNA binding assays

Binding reactions were conducted by mixing nucleic acids and proteins in the binding buffer (a commercial resource from Beyotime), in a final volume of 20 µl. The mixtures were kept at room temperature for 30 min and then separated on 0.7% agarose gels in TBE buffer. The mobility of the DNAs was detected by visualization of ethidium bromide stained gels and the [α-^32^P]dATP end labeled BSCTV DNA was detected by a PhosphorImager. All experiments were repeated a minimum of three times.

### Protoplast replication assay

Mesophyll protoplasts were prepared from rosette leaves of four-week-old Arabidopsis and were transfected as previously described [45]. Approximately 3×10^6^ protoplasts were transfected with ∼100 µg of the plasmid pCambia-1300-BSCTV, pCambia-1300-BSCTV-m1 and pCambia-1300-BSCTV-m2 independently extracted using a Plasmid Maxprep Kit (Vigorous Biotechnology). The transfected protoplasts were diluted with 8 ml of growth medium and kept at room temperature in the dark. Approximately 3×10^5^ cells were harvested at 0 and 4d after transfection and the total genomic DNA was extracted as described [46]. Viral DNA was identified with DNA gel blot analysis.

### Transient expression in *N. benthamiana* and Arabidopsis protoplasts

*N. benthamiana* leaves were infiltrated as described [47] with *A. tumefaciens* cells containing the pBAL-GFP vector constructed with PCR amplified C4 fused with GFP at the N-terminus under the control of 35S promoter. Leaves were imaged at 3d post infiltration by confocal microscopy. Arabidopsis mesophyll protoplasts were prepared as described above and 10^4^ protoplasts in 100 µl growth medium were transfected with 10 µg pGFP2-C4 vector in which C4 also fused with GFP at the N-terminus under the control of 35S promoter. The transfected protoplasts were diluted with 1 ml growth medium and were imaged using confocal microscope after being kept at room temperature in the dark for 16 hours.

### Confocal microscopy

Leaves and protoplasts images were captured by a Leica TCS SP5 confocal microscopy with an Argon ion laser and Leica TCS software. GFP fluorescence was excited at 488 nm and the emitted light was captured at 500 to 530 nm.

### Cell fraction assay and protein gel blot assay

GFP and GFP-C4 fusion proteins were transiently expressed in *N. benthamiana* leaf cells as referred above. Protein extraction for total, soluble and microsomal fractions were done as previously described [48]. Protein gel blot assay was performed according to the Clontech standard procedures with anti-GFP antibodies (JL-8). Secondary goat anti-mouse antibody conjugated to horseradish peroxidase was used and chemiluminescence was used for visualization as instructed by the manufacturer (ECL; Amersham Pharmacia).

### Construction of transgenic plants and the growth conditions

To produce C4 trangenic plants, an *Xho*I-*Spe*I fragment containing the *C4* cDNA was cloned into the vector pER8 in which transgene expression is under the control of an inducible XVE promoter which can be induced by estrogen [37]. Transformation of Arabidopsis was performed using *A. tumefaciens* strain EHA105 by vacuum infiltration method [49]. T3 homozygous lines were used for virus inoculation. *C4* transgenic plants were agro-inoculated with BSCTV and C4 deficient BSCTV mutants after grown for four weeks. *C4* expression was immediately induced by watering 2 µMβ-estradiol (E2758, Sigma) once two days after inoculation.

### PCR Amplification

To examine the present of BSCTV mutants in newly emerged leaves of C4 transgenic Arabidopsis, DNA was extracted and PCR amplification was done using forward (Fw, 5′-ATGCCTTTTTACAAAAAAGCC-3′) and reverse (Rev, 5′- TTACAAGGAAGTTTGATCTTG -3′) primers. After 30 PCR cycles with primer annealing temperature at 54°C, the fragments were separated on a 1% (w/v) agarose gel and recovered with DNA Gel Extraction Kit (Axygen Biosciences) for sequencing.
